# Difficult Diagnosis of Colon Adenocarcinoma Metastasis to Retina: A Case Report and Literature Review

**Published:** 2016-07-01

**Authors:** Ratnam Nookala, Vera V Batchu, Heather M Lee, Amir Loghmani, Gurdeep S Chhabra

**Affiliations:** 1 Department of Hematology-Oncology, Prince George’s Medical Center, Cheverly, Maryland, USA; 2 Radiation Oncology, Doctors Regional Cancer Center, Lanham, Maryland, USA

**Keywords:** Colorectal neoplasm, Metastasis to retina, Retinal metastasis, Eye neoplasm

## Abstract

Intraocular metastatic tumors have been increasingly reported in the recent past. Unlike choroidal metastasis, metastasis to retina is very rare and so far has been reported in very few case reports only.

A 56 year-old male who presented with a history of adenocarcinoma of the cecum and underwent lap colectomy for the primary cecal tumor, received adjuvant chemotherapy for a year after surgery and decided to stop. He was also diagnosed with metastasis to liver and lung at this time. He presented with left eye pain, pressure and decreased vision suspicious for retinal metastasis from cecal primary lesion, 2 years after initial diagnosis. A mass of 5 x 10 mm was found on ophthalmoscopic examination and on ultrasound of the eye, in spite of normal results of MRI of the orbit. Palliative radiation therapy of the left eye resulted in decreased eye pressure and improved vision.

As retinal metastasis carries a poorer prognosis due to higher risk of spread to central nervous system, the diagnosis of retinal metastasis in case of gastrointestinal cancers patients who present with vision changes should be made urgently. These patients should be thoroughly investigated with a synergistic approach of opthalmoscopic examination, ultrasound of the eye along with other imaging modalities like MRI of the orbit and just not MRI of orbit. Immediate action in the form of surgical or radiation treatments of the metastatic tumors of the eye should be instituted early on for a better prognosis.

## Introduction

 Until recently intraocular metastasis has been considered as a rare form of intraocular tumor; however, it is becoming increasingly common.^1^ Choroidal metastases are more common than retinal metastasis and are commonly spread from breast (50% of cases) followed by lung cancer.^1^

Retinal metastasis is very rare and few cases have been reported so far. Majority of the retinal metastasis spreads from cutaneous melanoma; lung primaries in males and breast primaries in females constitute a minor category.^2^ In contrast to choroidal metastasis occurring in 7% of visceral/gastrointestinal cancers, cases of gastrointestinal primary tumors metastasizing to retina are exceedingly rare.^2^ Colon cancer as the primary source has been reported in few cases.

However, the colorectal cancer as the third common cancer in the United States and third leading cause of death of cancer in both genders warrants a special attention.^3^ We present a case report of retinal metastasis from colon adenocarcinoma and then review the few reported cases.

## CASE REPORT

 A cecal adenocarcinoma metastasizing to retina is described in this case report. A 56 year-old male with history of alcohol abuse was admitted to ICU at Prince George's Hospital Center (PGH) on 05/12/2013 with severe symptomatic anemia and found to be guaiac positive. He underwent colonoscopy and biopsy of the cecal polyp was consistent with well differentiated adenocarcinoma. He underwent laparoscopic assisted right colectomy on 05/19/2013. The pathology report of the resected colon was consistent with well differentiated adenocarcinoma with focal mucinous differentiation arising from the cecum and invading through the muscularis propria and focally into the sub serosa (size 3.7 cm). The proximal, distal circumferential and mesenteric margins were negative for invasive carcinoma. There was no lymphatic vascular invasion and no lymph node metastasis at the time of resection (all of 12 Lymph nodes were negative for malignancy). The pathologic staging of the primary colon cancer was T3 N0 Mx. He received adjuvant chemotherapy for primary colon cancer. In 01/2014 he was admitted to PGH for abdominal discomfort. He was evaluated in hospital for pancreatitis and further work up with MRCP on 01/16/2014 showed evidence of 3 lesions in liver and subsequent CT guided core biopsy of liver lesions showed metastatic mucinous adenocarcinoma consistent with colon cancer as the primary source. His PET-CT scan on 02/14/14 showed liver and lung metastases.

The patient was advised to continue chemotherapy. However, the patient decided to hold off further chemotherapy and considered alternate regimens such as integrative oncology at Cancer Centers of Philadelphia. As per the medical records, patient stopped chemotherapy on 05/2014. Of note his DNA testing was negative for K-RAS and BRAF.

**Figure 1:**
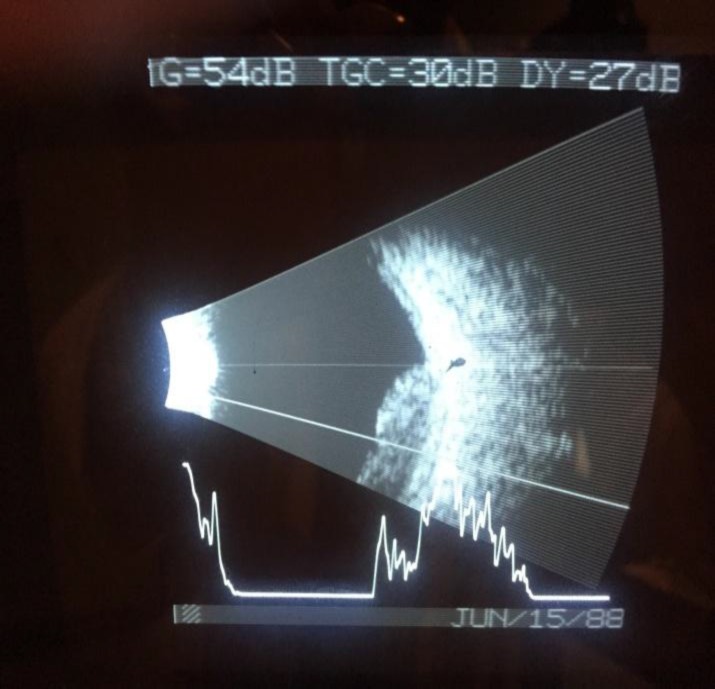
B-Scan of the left eye showing the retinal mass and fluid behind the retina. An elevated whitish mass with serous retinal detachment inferiorly. The mass measured 5 mm in elevation and 10 mm in diameter.

Patient was admitted in PGH from 03/15/15- 03/17/15 for productive cough. He was again advised to start chemotherapy; however, he opted to hold off on therapy till he explores all his options. On 06/24/2015 he was seen by the ophthalmologist for significant decrease in vision and pressure sensation in the left eye for couple of months which he described as a shadow along the supra temporal area in that eye. Dilated fundus examination of the left eye and the B-scan of the eye showed clear vitreous with normal optic nerve and just adjacent to the nerve an elevated whitish retinal mass with subsequent serous retinal detachment inferiorly. The mass measured 5 mm in elevation and 10 mm in diameter and extending up to the optic nerve, inferiorly up to the fovea and beyond the arcades (Figure 1).

**Figure 2:**
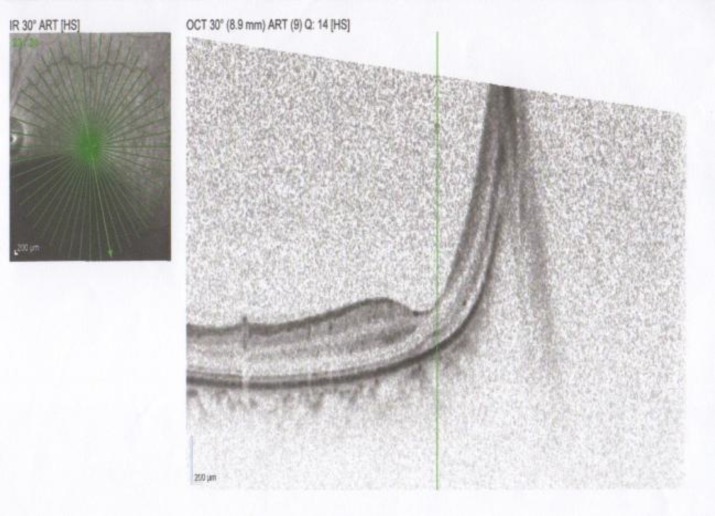
Optical Coherence Tomography (OCT) of the left eye showing retinal mass and fluid behind the retina with retinal detachment.

There was no hemorrhage and vitreoretinal traction. Subsequently he had an Optical Coherence Tomography of the left eye (Figure 2) and was found to have a retinal mass with fluid around the mass with retinal detachment. A high suspicion of colon cancer metastasis to eye was made by the ophthalmologist who carries a poor prognosis both for the eye and systemically since he would be at risk for CNS metastasis.

By this time he also had hepatic decompensation with ascites requiring paracentesis and DVT treated with Lovenox. He was sent to ED on 07/08/2015 for evaluation of the retinal tumor and for further work up and management. The inpatient MRI of the orbit was negative for any mass in the eye and orbit. He was referred to Radiation Oncologist for further course of action.

He was seen by radiation oncologist on 07/09/2015. Given his existing metastatic disease burden, the recommendation was made to give a course of palliative radiation therapy to the left eye.

**Figure 3:**
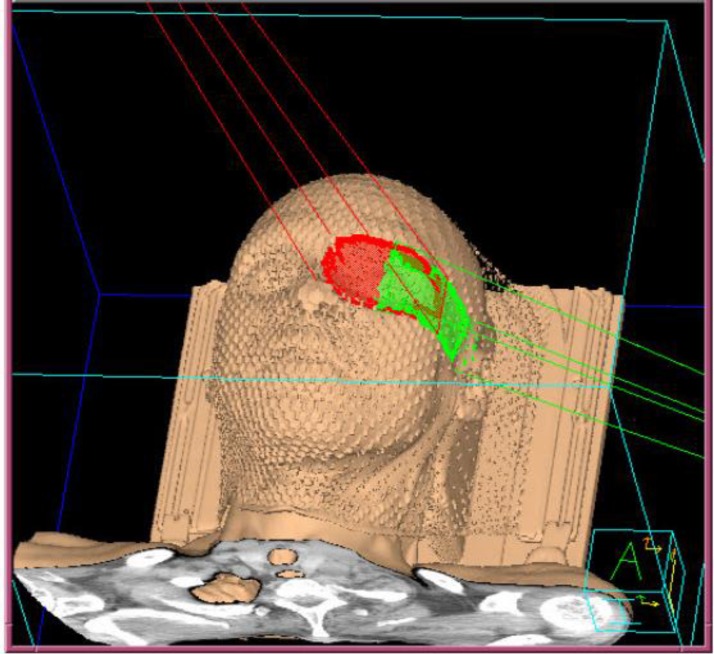
3-D image of CT simulation of the left orbit for radiation therapy

**Figure 4:**
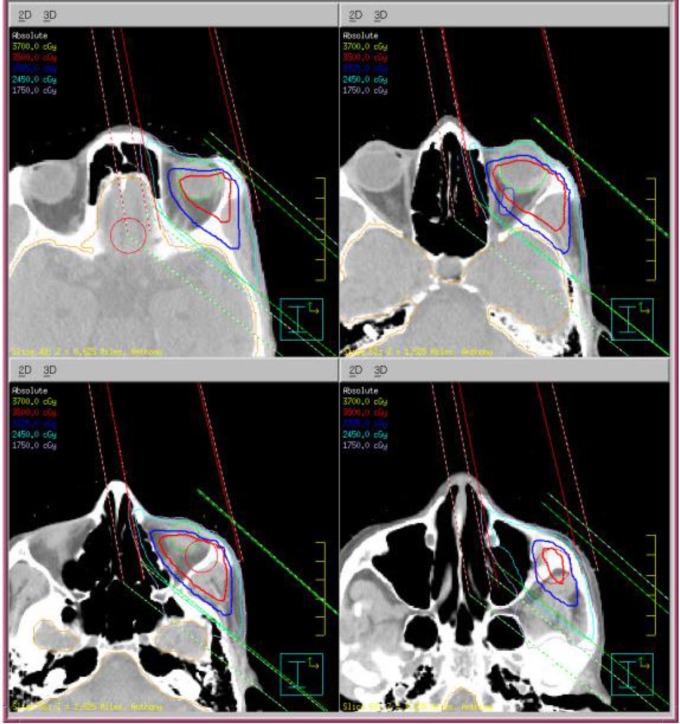
CT simulation of the left orbit showing the target site and the treatment plan for palliative radiation therapy. The patient received 3500 cGy of radiation in 14 fractions (250 cGy per fraction, 5 days a week)

The patient underwent CT simulation as part of the initial work up to develop a treatment plan and precisely identify and localize the treatment target (Figures 3 and 4). He is now receiving radiation to the tumor site with 3500 cGy in 14 fractions (250 cGy per fraction, 5 days a week) and then will be reevaluated.

## Discussion

 The current case presents an uncommon site of metastasis for colorectal cancer. Historically intraocular metastasis was considered a rare intraocular tumor; though recently it is considered more common.^1^ Unlike metastasis to choroid, retinal metastasis is very rare.^1^

In contrary to choroidal metastasis which occur 7% from visceral or gastrointestinal cancers,^2^^,^^4^^,^^5^ retinal metastasis from esophageal and pancreatic cancers are reported in few case reports. Utilizing OVID, PUBMED/MEDLINE and Google Scholar, we conducted a literature search to find out prior reported cases of retinal metastasis originating from colon cancer. Keywords used for the literature search included ‘colorectal neoplasms’, ‘metastasis to retina’/’retinal metastasis’ and ‘eye neoplasms’. The retinal metastasis from colon adenocarcinoma of the colon are reported in only 3 cases.^4^^,^^7^^,^^8^ First case was described by Kennedy et al.^3^ in 1956 in a 51 year-old male patient who presented with blurry vision in the right eye and on ophthalmoscopic examination was found to have 1/6 dd. Sized well circumscribed grayish white lesion in the macula extending into the vitreous. His past medical history was significant for intermittent rectal bleeding and underwent fistulectomy. He was referred to a proctologist who confirmed that the patient had annular carcinoma of the rectosigmoid junction. The patient immediately underwent rectosigmoidectomy and the pathology was consistent with adenocarcinoma of the rectosigmoid.

The follow-up ophthalmoscopic examination in 6 months from his initial visit showed increasing size of the tumor and new vessels coursing over the tumor prompting enucleation of the eye. The histopathology of the sections of the eye was consistent with that of the adenocarcinoma of the rectosigmoid. However, 3 months later the patient died most likely from metastatic disease and necropsy was denied. A second case was reported in a 74 year old woman with Muir-Torre syndrome; an autosomal dominant condition with skin lesions associated with visceral cancers.^6^ The patient had multiple tumors including sebaceous adenomas of face and neck status post excision, uterine leiomyoma, keratoacanthoma of the eyelid, squamous cell carcinoma of the forehead, adenocarcinoma of the breast status post mastectomy, adenocarcinoma of the colon and retinal tumor. The patient underwent enucleation of the eye for retinal tumor and the histopathology was consistent with adenocarcinoma. However, the source of primary tumor was not verified because of the unavailability of the breast adenocarcinoma samples for review.

Most recently another case of adenocarcinoma of the cecum metastasizing to retina was described by Apte et al.^7^ A 39 year-old male with adenocarcinoma of the cecum metastasizing to liver and lung underwent hemicolectomy. Six weeks after surgery, he received postoperative chemotherapy for 6 weeks and presented with complaints of vision changes in his left eye. Ophthalmologic examination revealed sub retinal hemorrhage in the eye. No mass was identified at that time. However, during his follow-up visit at 2.5 months, he was found to have a 2.8 mm x 5.3 mm x 6.6 mm retinal mass associated with exudative retinal detachment also involving the fovea. One month later, on dilated eye exam and ultrasonography, the mass was found to have increased in size significantly. He was still receiving chemotherapy for the primary cancer and underwent 3-port plana vitrectomy for the retinal tumor.^7^ The procedure took care of the entire retinal tumor en bloc due to confinement of the tumor to retina and not involving any surrounding structure. The histopathology of the tumor was consistent with the mucin-secreting adenocarcinoma of the colon primary. The patient received post-operative palliative radiation to the orbit and at 3 months follow-up visit he had a non-painful eye with normal intraocular pressure. His peripheral vision was regained subsequent to retinal reattachment procedure.^7^

When there is cancer metastasis to retina there is a high probability of the cancer spreading to central nervous system which carries poor prognosis. Hence, retinal metastasis should always be considered in the differential diagnosis of any colon cancer patient presenting with vision changes. Also, as demonstrated by our case, MRI of the orbit may not always be conclusive in diagnosing retinal tumors.

## CONCLUSION

 Clinical presentation of vision changes in a patient with history of colon/GI cancers should raise high index of suspicion for retinal metastasis and has to be evaluated by ophthalmoscopic examination involving dilated fundus exam, B-scan and OCT to arrive at a diagnosis and further assist in the treatment plan which is most often palliative radiation to the eye.^9^ This case forms a basis for difficult diagnosis of a rare clinical presentation.
